# Benign Paroxysmal Positional Vertigo and the Increased Risk of Ischemic Stroke: A Nested Case-Control Study Using a National Cohort Sample

**DOI:** 10.1155/2021/6629028

**Published:** 2021-02-20

**Authors:** Hyo Geun Choi, So Young Kim

**Affiliations:** ^1^Department of Otorhinolaryngology-Head & Neck Surgery, Hallym University College of Medicine, Anyang, Republic of Korea; ^2^Department of Otorhinolaryngology-Head & Neck Surgery, CHA Bundang Medical Center, CHA University, Seongnam, Republic of Korea

## Abstract

A recent population cohort study reported that benign paroxysmal positional vertigo (BPPV) was a risk factor for ischemic stroke. This study investigated the risk of ischemic and hemorrhagic strokes in patients with BPPV. A nested case-control study used the data from the Korean National Health Insurance Service-National Sample Cohort between 2002 and 2013. We used data of patients aged ≥50 years obtained from the Korean National Health Insurance Service-National Sample Cohort between 2002 and 2013. A total of 15,610 patients with ischemic stroke and 4,923 patients with hemorrhagic stroke were matched for age, sex, income, residential location, hypertension, diabetes, and dyslipidemia with 62,440 and 19,692 controls, respectively. History of BPPV was evaluated in the stroke and control groups. Crude and adjusted odds ratios (ORs) for stroke in patients with BPPV were analyzed using stratified logistic regression analysis. Subgroup analyses were performed for age and sex. Notably, 3.7% (572/15,610) of patients with ischemic stroke and 2.7% (1,702/62,440) of the control subjects reported a history of BPPV (*P* < 0.001). The adjusted OR for BPPV in patients with ischemic stroke was 1.35 (95% confidence interval (CI) 1.22–1.49, *P* < 0.001). Patients with ischemic stroke showed higher ORs for BPPV in the subgroup of women. Patients with hemorrhagic stroke did not show a high OR for BPPV. Ischemic stroke patients demonstrated the increased OR for BPPV in subjects aged ≥50 years old.

## 1. Introduction

Benign paroxysmal positional vertigo (BPPV) is a common cause of vertigo. A study that investigated German patients reported that the prevalence of BPPV was 2.4%, and BPPV accounted for moderate-to-severe dizziness in 8% of the patients investigated [1]. Previous studies have reported that the prevalence of BPPV increases with age [2]. Dislodgement of otoconia from the macula of the utricle or saccule produces symptoms of vertigo causing BPPV, and this condition is typically aggravated by positional changes [3]. BPPV is a benign condition, and patients do not usually present with systemic issues [4, 5]. However, recent studies have reported a high prevalence of chronic diseases, including hypertension, coronary heart disease, and diabetes, in patients with BPPV [6–8]. Additionally, a few studies have suggested that vertebrobasilar ischemia can induce dizziness and inner ear disorders that include BPPV [9–11].

Strokes are a major cause of mortality and morbidity worldwide. Globally, approximately 6,500,000 deaths and 113,000,000 disability-adjusted life-years are attributable to strokes [12]. In South Korea, the stroke incidence rate is 216 per 100,000 person-years, and the stroke prevalence rate is 15.9 per 1,000 persons [13]. Early diagnosis and intervention are crucial for successful treatment in patients with acute ischemic stroke because prompt thrombolytic treatment improves outcomes [14]. Several chronic diseases including hypertension, dyslipidemia, diabetes, and depression serve as risk factors for stroke [15, 16]. A recent population cohort study reported that BPPV was a predictor of ischemic strokes [7]; however, patients with ischemic stroke included in the study showed a high prevalence of chronic diseases, which might serve as confounders. Although the potential confounders were adjusted in the study, only age and sex were matched between the ischemic stroke and control groups. Matching the control group for medical history is crucial because it may influence the association between independent and dependent variables [17].

We hypothesized that BPPV increases the risk of ischemic stroke even after matching and adjusting for past medical history, and we investigated a large population-based cohort of patients aged ≥50 years to address this hypothesis. Strokes are rare in young individuals and are usually associated with a specific underlying etiology. Therefore, we selected older patients for our study, although previous studies investigating BPPV used patients aged ≥20 years [7]. We performed subgroup analyses to investigate the age- and sex-specific risks of stroke in patients with BPPV. Because hemorrhagic stroke is one of the causes of vertigo attack and has common risk factors [18, 19], we also separately investigated the association between BPPV and hemorrhagic strokes.

## 2. Materials and Methods

### 2.1. Statement of Approval of the Institution's Ethics Committee

The Hallym University Ethics Committee approved the use of the study data (approval protocol number: 2017-I102). The Institutional Review Board waived the need for written informed consent for this study.

This national cohort study used data from the Korean National Health Insurance Service (NHIS)-National Sample Cohort. Details regarding this data have been described in our previous studies [20, 21].

**Figure 1:**
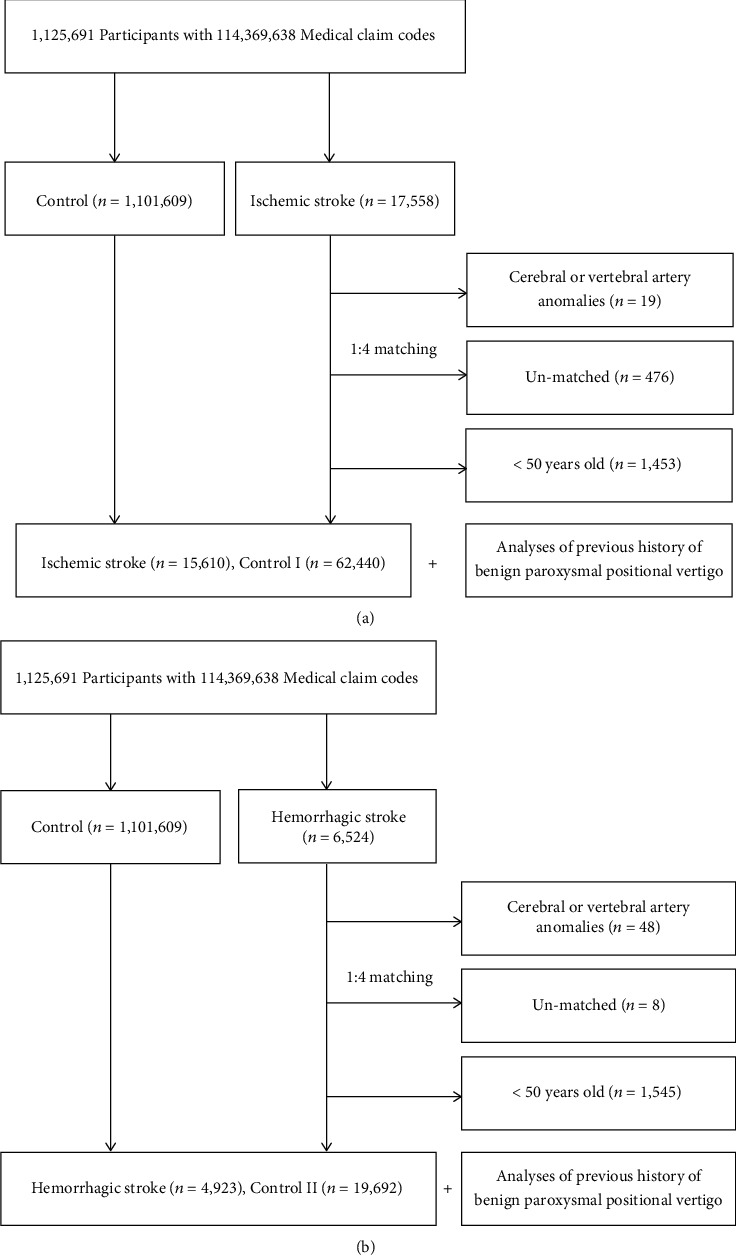
Schematic illustration of the participant selection process used in this study. (a) From a total of 1,125,691 participants, 15,610 ischemic stroke participants were matched with 62,440 control I participants for age, group, sex, income group, residential location, and past medical history. (b) From a total of 1,125,691 participants, 4,923 hemorrhagic stroke participants were matched with 19,692 control II participants for age, group, sex, income group, residential location, and past medical history.

Patients with ischemic and hemorrhagic stroke were matched in a ratio of 1 : 4 with control group subjects who were not diagnosed with a hemorrhagic or ischemic stroke between 2002 and 2013. The control groups were selected from the parent population (*n* = 1,101,609). The matches were processed for age, sex, income, residential location, and medical history (hypertension, diabetes, and dyslipidemia). To prevent a selection bias when matching subjects, control group subjects were sorted randomly and were selected from top to bottom. The control group subjects were matched with patients with stroke for the index date (diagnosed date of their matched stroke). Therefore, control group subjects who died before the index date were excluded. The following patients were excluded from the study: patients with cerebral or vertebral anomalies (Q28) (*N* = 19 with ischemic stroke and *N* = 48 with hemorrhagic stroke), patients with stroke for whom a sufficient number of matching subjects could not be identified (*N* = 476 for patients with ischemic stroke and *N* = 8 for patients with hemorrhagic stroke), and patients aged <50 years (*N* = 1,453 for patients with ischemic and *N* = 1,545 for patients with hemorrhagic stroke). Finally, 1 : 4 matching resulted in the inclusion of 15,610 patients with ischemic and 4,923 patients with hemorrhagic stroke and 62,440 subjects in control group I and 19,692 subjects in control group II (Figure 1). Notably, patients were not matched strictly for history of ischemic heart disease and depression because this would increase the drop-out rate secondary to the lack of an adequate number of subjects in the control group. After matching, we obtained information regarding a previous history of BPPV in both, the stroke and the control groups.

### 2.2. Selection of Participants

Among a total of 1,125,691 cases with 114,369,638 medical claim codes, we included patients with a diagnosis of hemorrhagic or ischemic strokes. Hemorrhagic (I60–I62) and ischemic (I63) strokes were diagnosed using the International Classification of Diseases, Tenth Revision, Clinical Modification (ICD-10) codes. We selected patients who had been treated for stroke at least once. This method has been used to evaluate the stroke incidence rate in previous South Korean studies [13, 22]. A total of 17,558 patients with ischemic and 6,524 patients with hemorrhagic stroke were identified between 2002 and 2013.

BPPV was diagnosed using the ICD-10 codes (H811) for cases recorded between 2002 and 2013. Among these cases, we only included patients who had been treated at least twice.

### 2.3. Variables

Patients were grouped by age at 5-year intervals into 8 groups as follows: 50–54, 55–59, 60–64, 65–69, 70–74, 75–79, 80–84, and ≥85 years. Patients were categorized into 41 income groups (1: health aid class, 20: self-employed health insurance class, and 20: employed health insurance class). These groups were recategorized into 5 subclasses, with class 1 = the lowest income and class 5 = the highest income. Residential locations were categorized into 16 regions based on their administrative center. These regions were recategorized as urban (Seoul, Busan, Daegu, Incheon, Gwangju, Daejeon, and Ulsan) and rural (Gyeonggi, Gangwon, Chungcheongbuk, Chungcheongnam, Jeollabuk, Jeollanam, Gyeongsangbuk, Gyeongsangnam, and Jeju) regions.

The past medical histories of patients were evaluated based on the ICD-10 codes. To ensure accurate diagnoses, patients with hypertension (I10 and I15), diabetes (E10–E14), and dyslipidemia (E78) were defined as those who were treated for these conditions at least twice. Patients with ischemic heart disease (I24 and I25), peripheral vascular disease (I71, I790, I739, R02, Z958, and Z959), and atrial fibrillation and flutter (I48) were defined as those who were treated at least once. Patients with depression were defined as those in whom depression was diagnosed by a psychiatrist on ≥2 occasions based on ICD-10 codes for bipolar affective disorder (F31) through unspecified mood disorder (F39). Patients with chronic obstructive pulmonary disease (COPD) were defined as those diagnosed with emphysema (J43) through other COPD (J44) and were treated with short- and long-acting beta2-agonists, long-acting muscarinic antagonists, and corticosteroids [23].

### 2.4. Statistical Analysis

Chi-squared tests were used to compare general characteristics between the stroke and control groups. Unconditional logistic regression analysis was performed to analyze the odds ratios (ORs) for BPPV in patients with stroke. A crude (simple) model and a model adjusted for ischemic heart disease, peripheral vascular disease, atrial fibrillation and flutter, depression, and COPD were used for analysis. Age, sex, income, and residential location were stratified. Additionally, 95% confidence intervals (CIs) were calculated. Additionally, we analyzed the interaction of age∗sex in the logistic regression model (Table S1). As the age∗sex were statistically significant variables, we added subgroup analyses according to age and sex. For subgroup analyses, patients were categorized on the basis of age and sex (<70 years, ≥70 years, men, and women). Two-tailed tests were used, and a *P* value < 0.05 was considered statistically significant. All data were analyzed using the SPSS software, ver. 22.0 (IBM, Armonk, NY, USA).

## 3. Results

We observed that 3.7% (572/15,610) of the patients with ischemic stroke reported a history of BPPV, which was higher than that observed in the control I group (2.7% (1,702/62,440); *P* < 0.001, Table 1), and of the patients with hemorrhagic stroke, 2.7% (132/4,923) of the stroke group and 2.4% (473/19,692) of the control II groups reported a history of BPPV (*P* = 0.258). The 25.2% (95%CI = 23.4–27.0) and 19.9% (95%CI = 19.6–20.1) of BPPV and non-BPPV participants had ischemic stroke (*P* < 0.001, Table S2). The 21.8% (95%CI = 18.6–25.3) and 20.0% (95%CI = 19.5–20.5) of BPPV and non-BPPV participants had hemorrhagic stroke (*P* = 0.258, Table S2). The stroke and control groups were matched for age, sex, income, residential location, and medical history of hypertension, diabetes, and dyslipidemia. Ischemic heart disease, depression, and atrial fibrillation and flutter were more prevalent in the ischemic stroke group than in the control I group (both *P* < 0.001). However, the prevalence rates of ischemic heart disease and depression were comparable with the control II group in the hemorrhagic stroke group. The prevalence of atrial fibrillation and flutter was higher in the hemorrhagic stroke group (*P* < 0.001). The prevalence of peripheral vascular disease was lower in the ischemic stroke and hemorrhagic stroke groups compared to those of control groups.

The crude OR for BPPV was higher in the ischemic stroke group than in the control I group (OR 1.36, 95% CI 1.24–1.50, *P* < 0.001, Table 2). The OR for BPPV remained higher in the ischemic stroke group than in the control I group even after adjusting for ischemic heart disease, peripheral vascular disease, and atrial fibrillation and flutter, depression, and COPD (adjusted OR 1.35, 95% CI 1.22–1.49, *P* < 0.001). In the age/sex subgroup analyses, the group comprising women aged <70 years showed higher ORs for BPPV in the ischemic stroke group (adjusted OR 1.83, 95% CI 1.47–2.26, *P* < 0.001, Table 3). The group comprising women aged ≥70 years showed an OR of 1.29 for BPPV in the ischemic stroke group (95% CI 1.11–1.49, *P* < 0.001).

Patients with hemorrhagic stroke only show a high OR for BPPV in ≥70 years old women (adjusted OR 1.34, 95% CI 1.00-1.79, *P* = 0.048).

## 4. Discussion

The risk of ischemic stroke was higher in patients with BPPV than in the control group subjects matched and adjusted for age, sex, income, residential location, and past medical history. The women's subgroup showed a higher OR for BPPV in patients with ischemic stroke. In contrast, the OR for BPPV was not high in patients with hemorrhagic stroke. This study was an extension of previous studies and included a large population cohort and matched control groups. Additionally, few previous studies have evaluated and differentiated between the risks of ischemic and hemorrhagic stroke in patients with BPPV.

Previous studies investigating the association between BPPV and stroke have reported conflicting results. A previous study reported a higher risk of ischemic stroke in patients with BPPV [7], which concurs with our study. The risk of ischemic stroke was 1.42-fold higher in patients with BPPV than in the control group after adjusting for age, sex, and past medical history (95% CI 1.16–1.73, *P* = 0.001) [7]. However, another population-based study reported that only 0.7% (9/1,297) of patients with an isolated episode of dizziness developed a stroke or transient ischemic attack [24]. This particular study did not compare between patients with dizziness and a control group nor did it stratify patients with dizziness based on their diseases. No previous studies have investigated the association between hemorrhagic stroke and BPPV.

Physical inactivity following BPPV might increase the risk of an ischemic stroke. Episodes of vertigo tend to recur, and anxiety may be associated with dizziness; therefore, patients with BPPV may be less physically active following an episode of BPPV. Notably, physical activity scores were lower in patients with BPPV than in control subjects, particularly in those aged >60 years [25]. Physical inactivity can increase the risk of stroke. A previous study has reported that physical inactivity increased the risk of stroke in middle-aged and older adults (OR 1.74, 95% CI 1.16–2.59) [26]. Previous studies have shown indirect associations between these factors in that a sedentary lifestyle increased the risk of obesity, cardiovascular disease, and diabetes [27, 28], and these chronic diseases are known to contribute to stroke onset [29]. Although this study matched and adjusted control subjects with respect to these chronic diseases, indirect effects could alter the risk of ischemic stroke in patients with BPPV.

Ischemic changes affecting the vestibular artery in patients with BPPV could precede a full-blown ischemic stroke. Several previous studies have suggested that ischemic vascular changes may contribute to the pathophysiology of BPPV [7, 30]. The vascular supply to the vestibular system originates from the anterior inferior cerebellar artery, which branches into the anterior vestibular artery. Owing to the limited collateral supply to the vestibular system, it is vulnerable to ischemic obstruction. Therefore, ischemic changes affecting the vertebrobasilar system could initially produce vestibular symptoms, such as BPPV. A few retrospective studies have reported that the prevalence of cardiovascular diseases, including hypertension, was higher in patients with BPPV than in control groups [6, 7]. Moreover, the carotid artery intima-media thickness is known to be greater in patients with BPPV than in patients with other peripheral vestibular disorders [31]. In addition to the mechanical pathophysiology explained by displacement of otoconia, the aforementioned atherosclerotic changes observed in patients with BPPV indicate the role of a vascular etiology in patients with BPPV.

Common risk factors associated with both, BPPV and ischemic stroke, contribute to the increased risk of ischemic stroke in patients with BPPV. Although we matched and adjusted for several potential confounders in this study, an unknown factor could increase the risk of both, ischemic stroke and BPPV. For example, previous research has indicated that osteoporosis increased the risks of both, stroke and BPPV [32, 33].

In this study, age/sex subgroup analyses showed that the risk of ischemic stroke was higher in the subgroup of women with BPPV. The higher prevalence of BPPV in women might affect the statistical likelihood of a higher risk of ischemic stroke in these patients with BPPV [34]. Additionally, other risk factors associated with ischemic stroke, such as smoking habits and alcohol consumption, are more commonly associated with men than women. The ORs for ischemic stroke in patients with BPPV were higher in subgroups with patients aged <70 years than in subgroups with patients aged ≥70 years. Other comorbidities might affect the risk of ischemic stroke in older patients secondary to the higher prevalence of these comorbidities in older patients. A previous study reported that labyrinthine hemorrhage could induce BPPV via the action of residual debris from the blood in the endolymphatic fluid [35]. However, hemorrhagic stroke was not associated with BPPV in this study. The small percentage of hemorrhagic stroke observed might explain the lack of association between BPPV and hemorrhagic stroke. However, the prevalence rates of different types of stroke differ across countries in that approximately 80% of strokes were reportedly ischemic strokes, per an Asian survey [36].

This study is based on nationwide representative data that were validated using a previous study [37]. Because the NHIS data include all citizens of the nation without exception, no patients were missing in the study cohort. The control group was randomly selected and matched for age, sex, income, residential location, and past medical history of hypertension, diabetes, and dyslipidemia. Income levels and residential location are important determinants of access to medical care; therefore, these variables were matched and adjusted using the NHIS data. The BPPV classification was based on physicians' diagnoses and a history of receiving treatments on ≥2 occasions. However, it is possible that positional nystagmus of central origin can also manifest as BPPV [38]. Approximately 97.5% of central nystagmus reportedly occurs in an atypical direction during the Dix–Hallpike maneuver; however, if central nystagmus is caused by cerebellar or brainstem pathology, it may be misinterpreted as BPPV [38]. The potential misinterpretation of cerebellar or brainstem pathology as BPPV might overestimate the association of BPPV with stroke in the present study. However, this was attenuated by excluding the participants with cerebral or vertebral artery anomalies and adjusted ischemic/hemorrhagic stroke and peripheral vascular diseases.

Additional limitations of this study are as follows: although we adjusted for several confounders, we did not consider a few variables, including body mass index, smoking status, and alcohol consumption. Reportedly, smoking shows a negative association with BPPV [39]. To attenuate the possible confounding effect of smoking habits, COPD was added and adjusted as a variable for analysis in this study. The study drop-out rate increased with the number of matching variables; thus, these variables were limited to ensure that the study population was representative of the South Korean population. In this study, stroke groups were diagnosed using ICD-10 codes. However, the Health Insurance Review and Assessment Service National Patient Sample data did not include information on the severity and radiological confirmation regarding the site of each stroke. For example, anterior vs. posterior circulation strokes were not differentiated. Similarly, details of the semicircular canals affected by BPPV and information regarding patient recovery were unavailable.

## 5. Conclusion

Ischemic stroke increased the OR of BPPV in patients aged ≥50 years. The OR for BPPV in patients with ischemic stroke was higher in subgroups comprising women. Hemorrhagic stroke did not increase the OR for BPPV.

## Figures and Tables

**Table 1:** General characteristics of participants.

Characteristics	Ischemic stroke			Hemorrhagic stroke		
	Ischemic stroke (*n*, %)	Control I group (*n*, %)	*P* value	Hemorrhagic stroke (*n*, %)	Control II group (*n*, %)	*P* value
Age (years old)						
50-54	1,121 (7.2)	4,484 (7.2)		656 (13.3)	2,624 (13.3)	
55-59	1,390 (8.9)	5,560 (8.9)		619 (12.6)	2,476 (12.6)	
60-64	1,926 (12.3)	7,704 (12.3)		714 (14.5)	2,856 (14.5)	
65-69	2,537 (16.3)	10,148 (16.3)		771 (15.7)	3,084 (15.7)	
70-74	3,027 (19.4)	12,108 (19.4)		791 (16.1)	3,164 (16.1)	
75-79	2,795 (17.9)	11,180 (17.9)		674 (13.7)	2,696 (13.7)	
80-84	1,765 (11.3)	7,060 (11.3)		402 (8.2)	1,608 (8.2)	
85+	1,049 (6.7)	4,196 (6.7)		296 (6.0)	1,184 (6.0)	
Sex						
Male	7,653 (49.0)	30,612 (49.0)		2,379 (48.3)	9,516 (48.3)	
Female	7,957 (51.0)	31,828 (51.0)		2,544 (51.7)	10,176 (51.7)	
Income						
1 (lowest)	3,295 (21.1)	13,180 (21.1)		1,005 (20.4)	4,020 (20.4)	
2	1,935 (12.4)	7,740 (12.4)		654 (13.3)	2,616 (13.3)	
3	2,342 (15.0)	9,368 (15.0)		781 (15.9)	3,124 (15.9)	
4	3,019 (19.3)	12,076 (19.3)		996 (20.2)	3,984 (20.2)	
5 (highest)	5,019 (32.2)	20,076 (32.2)		1,487 (30.2)	5,948 (30.2)	
Region of residence						
Urban	6,251 (40.0)	25,004 (40.0)		2,045 (41.5)	8,180 (41.5)	
Rural	9,359 (60.0)	37,436 (60.0)		2,878 (58.5)	11,512 (58.5)	
Hypertension	12,559 (80.5)	50,236 (80.5)		3,534 (71.8)	14,136 (71.8)	
Diabetes	6,429 (41.2)	25,716 (41.2)		1,367 (27.8)	5,468 (27.8)	
Dyslipidemia	5,791 (37.1)	23,164 (37.1)		1,259 (25.6)	5,036 (25.6)	1.000
Ischemic heart disease	2,555 (16.4)	8,456 (13.5)	<0.001^∗^	530 (10.8)	2,181 (11.1)	0.535
Depression	2,058 (13.2)	6,968 (11.2)	<0.001^∗^	537 (10.9)	2,144 (10.9)	0.967
Peripheral vascular disease	4,791 (30.7)	19,805 (31.7)	0.012^∗^	1,078 (21.9)	5,607 (28.5)	<0.001^∗^
Atrial fibrillation and flutter	1,652 (10.6)	3,004 (4.8)	<0.001^∗^	336 (6.8)	785 (4.0)	<0.001^∗^
COPD	2,742 (17.6)	10,921 (17.5)	0.825	179 (15.2)	3,667 (15.7)	0.687
BPPV	572 (3.7)	1,702 (2.7)	<0.001^∗^	132 (2.7)	473 (2.4)	0.258

^∗^Chi-square test. Significance at *P* < 0.05. COPD: chronic obstructive pulmonary disease; BPPV: benign paroxysmal positional vertigo.

**Table 2:** Crude and adjusted odds ratios (95% confidence interval) of stroke for BPPV.

Characteristics	BPPV			
	Crude^†^	*P* value	Adjusted^†‡^	*P* value
Ischemic stroke	1.36 (1.24-1.50)	<0.001^∗^	1.35 (1.22-1.49)	<0.001^∗^
Control I	1.00		1.00	
Hemorrhagic stroke	1.12 (0.92-1.37)	0.254	1.18 (0.84-1.66)	0.343
Control II	1.00		1.00	

^∗^Conditional logistic regression analyses, significance at *P* < 0.05. ^†^Stratified for age, sex, income, region of residence, hypertension, diabetes, and dyslipidemia. ^‡^Adjusted model for chronic obstructive pulmonary disease, ischemic heart disease, peripheral vascular disease, atrial fibrillation and flutter, and depression.

**Table 3:** Subgroup analyses of crude and adjusted odds ratios (95% confidence interval) of stroke for BPPV according to age and sex.

Characteristics	BPPV			
	Crude^†^	*P* value	Adjusted^†‡^	*P* value
Age < 70 years old, men (*n* = 21,100)				
Ischemic stroke	1.34 (1.04-1.73)	0.026^∗^	1.29 (0.99-1.67)	0.057
Control I	1.00		1.00	
Age < 70 years old, women (*n* = 13,770)				
Ischemic stroke	1.87 (1.51-2.31)	<0.001^∗^	1.83 (1.47-2.26)	<0.001^∗^
Control I	1.00		1.00	
Age ≥ 70 years old, men (*n* = 17,165)				
Ischemic stroke	1.15 (0.92-1.44)	0.227	1.15 (0.92-1.44)	0.222
Control I	1.00		1.00	
Age ≥ 70 years old, women (*n* = 26,015)				
Ischemic stroke	1.28 (1.11-1.48)	<0.001^∗^	1.29 (1.11-1.49)	<0.001^∗^
Control I	1.00		1.00	
Age < 70 years old, men (*n* = 7,665)				
Hemorrhagic stroke	1.13 (0.64-1.97)	0. 679	1.13 (0.64-1.99)	0.672
Control II	1.00		1.00	
Age < 70 years old, women (*n* = 6,135)				
Hemorrhagic stroke	0.85 (0.56-1.29)	0.443	0.91 (0.60-1.38)	0.660
Control II	1.00		1.00	
Age ≥ 70 years old, men (*n* = 4,230)				
Hemorrhagic stroke	1.20 (0.75-1.94)	0.445	1.24 (0.77-2.00)	0.383
Control II	1.00		1.00	
Age ≥ 70 years old, women (*n* = 6,585)				
Hemorrhagic stroke	1.27 (0.95-1.68)	0.108	1.34 (1.00-1.79)	0.048^∗^
Control II	1.00		1.00	

^∗^Conditional logistic regression analyses, significance at *P* < 0.05. ^†^Stratified for age, sex, income, region of residence, hypertension, diabetes, and dyslipidemia. ^‡^Adjusted model for chronic obstructive pulmonary disease, ischemic heart disease, peripheral vascular disease, atrial fibrillation and flutter, and depression.

## Data Availability

Releasing of the data by the researcher is not allowed legally. All data are available from the database of the National Health Insurance Sharing Service (NHISS). https://nhiss.nhis.or.kr/NHISS allows all of this data for any researcher who promises to follow the research ethics with some cost. If you want to access the data of this article, you could download it from the website after promising to follow the research ethics.

## Supplementary Materials

Supplementary 1 Table S1: crude and adjusted odds ratios (95% confidence interval) of stroke for BPPV with interaction of the age∗sex model.

Supplementary 2 Table S2: the percentages and 95% confidence intervals of ischemic stroke and hemorrhagic stroke in BPPV and non-BPPV.
