# Supplementary material for: Benign Paroxysmal Positional Vertigo and the Increased Risk of Ischemic Stroke: A Nested Case-Control Study Using a National Cohort Sample
Source: Biomed Res Int. 2021 Feb 20;2021:6629028. doi: 10.1155/2021/6629028 (PMC7924071; doi:10.1155/2021/6629028)
Supplement: Supplementary 1 — Table S1: crude and adjusted odds ratios (95% confidence interval) of stroke for BPPV with interaction of the age∗sex model. [file 6629028.f1.docx]

**Table S1** Crude and adjusted odd ratios (95% confidence interval) of stroke for BPPV with interaction of age*sex model

| Characteristics | | BPPV | | | | | |
| --- | --- | --- | --- | --- | --- | --- | --- |
|  |  | Crude† | P-value | Model 1†‡ | P-value | Model 2§ | P-value |
| Ischemic stroke | | 1.36 (1.24-1.50) | <0.001* | 1.35 (1.22-1.49) | <0.001* | 1.35 (1.23-1.49) | <0.001* |
| Age*sex | |  |  |  |  | 0.93 (0.89-0.98) | <0.001* |
| Age group | |  |  | 1.14 (1.11-1.16) | <0.001* | 1.27 (1.16-1.39) | <0.001* |
| Sex (ref = men) | |  |  | 1.61 (1.47-1.77) | <0.001* | 4.43 (2.08-9.43) | <0.001* |
| Control I | | 1.00 |  | 1.00 |  | 1.00 |  |
| Hemorrhagic stroke | | 1.12 (0.92-1.37) | 0.254 | 1.18 (0.84-1.66) | 0.343 | 1.20 (0.86-1.68) | 0.292 |
| Age*sex | |  |  |  |  | 0.94 (0.89-0.98) | 0.009* |
| Age group | |  |  | 1.13 (1.11-1.16) | <0.001* | 1.27 (1.16-1.38) | <0.001* |
| Sex (ref = men) | |  |  | 1.61 (1.47-1.77) | <0.001* | 4.39 (2.07-9.35) | <0.001* |
| Control II | | 1.00 |  | 1.00 |  |  |  |

* Conditional logistic regression analyses, Significance at P < 0.05

† Stratified for age, sex, income, region of residence, hypertension, diabetes, and dyslipidemia (except for age and sex as independent variables in model 1)

‡ Model 1 was adjusted for chronic obstructive pulmonary disease, ischemic heart disease, peripheral vascular disease, atrial fibrillation and flutter, and depression regarding stroke as independent variable. Other independent variables were adjusted for following covariates: age, sex, income, region of residence, ischemic stroke (or hemorrhagic stroke), hypertension, diabetes, dyslipidemia, chronic obstructive pulmonary disease, ischemic heart disease, peripheral vascular disease, atrial fibrillation and flutter, and depression.

§ Model 2 was adjusted for age, sex, interaction of age*sex, income, region, ischemic stroke (or hemorrhagic stroke), hypertension, diabetes, dyslipidemia, chronic obstructive pulmonary disease, ischemic heart disease, peripheral vascular disease, atrial fibrillation and flutter, and depression.
