# Supplementary material for: Benign Paroxysmal Positional Vertigo and the Increased Risk of Ischemic Stroke: A Nested Case-Control Study Using a National Cohort Sample
Source: Biomed Res Int. 2021 Feb 20;2021:6629028. doi: 10.1155/2021/6629028 (PMC7924071; doi:10.1155/2021/6629028)
Supplement: Supplementary 2 — Table S2: the percentages and 95% confidence intervals of ischemic stroke and hemorrhagic stroke in BPPV and non-BPPV. [file 6629028.f2.docx]

**Table S2** The percentages and 95% confidence intervals of ischemic stroke and hemorrhagic stroke in BPPV and non-BPPV

| BPPV and non-BPPV | | No. of stroke | % of stroke (95% CI) | P-value |
| --- | --- | --- | --- | --- |
| % of ischemic stroke | |  |  | <0.001* |
|  | BPPV (n = 2,274) | 572 | 25.1 (23.4-27.0) |  |
|  | Non-BPPV (n = 75,776) | 15,038 | 19.9 (19.6-20.1) |  |
| % of hemorrhagic stroke | |  |  | 0.258 |
|  | BPPV (n = 605) | 132 | 21.8 (18.6-25.3) |  |
|  | Non-BPPV (n = 24,010) | 4,791 | 20.0 (19.5-20.5) |  |

*Chi-square test. Significance at P < 0.05
